# A highly sensitive NanoLuc-based protease biosensor for detecting apoptosis and SARS-CoV-2 infection

**DOI:** 10.1038/s41598-023-28984-4

**Published:** 2023-01-31

**Authors:** Masashi Arakawa, Akiho Yoshida, Shinya Okamura, Hirotaka Ebina, Eiji Morita

**Affiliations:** 1 Department of Biochemistry and Molecular Biology, Faculty of Agriculture and Life Science, Hirosaki University, 3 Bunkyo-Cho, Hirosaki-Shi, Aomori, 036-8561 Japan; 2 Division of Biomolecular Function, Bioresources Science, United Graduate School of Agricultural Sciences, Iwate University, Morioka, 020-0066 Japan; 3 Virus Vaccine Group, BIKEN Innovative Vaccine Research Alliance Laboratories, Institute for Open and Transdisciplinary Research Initiatives, Osaka University, Suita, Osaka Japan; 4 Virus Vaccine Group, BIKEN Innovative Vaccine Research Alliance Laboratories, Research Institute for Microbial Diseases, Osaka University, Suita, Osaka Japan; 5 The Research Foundation for Microbial Diseases of Osaka University, Suita, Osaka Japan

**Keywords:** Protein design, SARS-CoV-2, Apoptosis

## Abstract

Proteases play critical roles in various biological processes, including apoptosis and viral infection. Several protease biosensors have been developed; however, obtaining a reliable signal from a very low level of endogenous protease activity remains a challenge. In this study, we developed a highly sensitive protease biosensor, named FlipNanoLuc, based on the *Oplophorus gracilirostris* NanoLuc luciferase. The flipped β-strand was restored by protease activation and cleavage, resulting in the reconstitution of luciferase and enzymatic activity. By making several modifications, such as introducing NanoBiT technology and CL1-PEST1 degradation tag, the FlipNanoLuc-based protease biosensor system achieved more than 500-fold luminescence increase in the corresponding protease-overexpressing cells. We demonstrated that the FlipNanoLuc-based caspase sensor can be utilized for the detection of staurosporine-induced apoptosis with sixfold increase in luminescence. Furthermore, we also demonstrated that the FlipNanoLuc-based coronavirus 3CL-protease sensor can be used to detect human coronavirus OC43 with tenfold increase in luminescence and severe acute respiratory syndrome-coronavirus-2 infections with 20-fold increase in luminescence by introducing the stem-loop 1 sequence to prevent the virus inducing global translational shutdown.

## Introduction

Limited proteolysis mediated by specific proteases plays an important role in various biological processes such as apoptosis signaling, induction of inflammation, and maturation of viral proteins. In apoptosis and inflammation, the activation of caspase family proteases plays a crucial role in rapid signal transduction, from stimulation to the activation of specific substrates, such as caspase-activated DNase and interleukin-1β (IL-1β). Site-specific proteolysis of these molecules by caspases separates the inhibitory domains of their precursors and generates the active forms of these proteins. Viral protein maturation is regulated by site-specific proteolysis. Translated viral polyprotein precursors are processed by viral proteases such as 3C-like protease (3CLpro) or papain-like protease (PLpro) in beta-coronaviruses, to form viral proteins^1^. Molecular biosensors that detect specific proteolysis can be useful tools for monitoring these biological events both in vitro and in vivo. To date, several protease biosensors have been developed using coupling proteases that recognize and cleave peptide sequences for signal readout, such as fluorescent resonance energy transfer, luciferase, or fluorescent proteins. These biosensors have been applied for the measurement of the activities of proteases that are essential for several biological processes, including trypsin^2–5^, caspase-3^6–10^, matrix metalloproteinases^11^, and cathepsin-B^12–15^, as well as various viruses, such as hepatitis A virus^16^, dengue virus^17^, hepatitis C virus^18^, human immunodeficiency virus^19^, calicivirus^20^, and coronaviruses^21–26^. However, the problem regarding the sensitivity of these biosensors remains a critical issue; this needs to be addressed to achieve more reliable signals from endogenous proteases that have very low expression levels.

NanoLuc luciferase (NanoLuc) is derived from *Oplophorus gracilirostris* and produces a glow-type luminescence; this enzyme exhibits high physical stability with a signal half-life > 2 h, and high sensitivity and specificity approximately 150-fold greater than that of the firefly (*Photinus pyralis*) and Renilla (*Renilla reniformis*) luciferases. Furthermore, NanoLuc luciferase was applied to protein-fragment complementation system known as NanoLuc Binary Technology (NanoBiT)^27^. In this study, we designed and developed a novel protease reporter based on NanoLuc to develop a highly sensitive biosensor for detecting specific protease activities associated with apoptosis and human pathogenic coronavirus infections.

## Methods

### Cells, viruses, and reagents

The 293 T and Vero cells were cultured in Dulbecco’s Modified Eagle’s medium (DMEM) (Nacalai, Item No. 08458-16C) supplemented with 10% fetal bovine serum (FBS) (Thermo Fisher Scientific, 24F6486K), 100 U/mL penicillin, and 100 μg/mL streptomycin at 5% CO_2_ and 37 °C. Human angiotensin converting enzyme 2 (hACE2)-expressing BHK cells were generated using the PiggyBac transposon system (SystemBiosciences). The *hACE2* gene was cloned into a PB514B-2 plasmid. BHK cells (JCRB9020) were co-transfected with PB514B-2/hACE2 and PB200A-1 and selected with 3 µg/mL puromycin (Gibco). These cells were cultured in a Minimum Essential Eagle Medium (Sigma-Aldrich) supplemented with 10% fetal bovine serum, 100 U/mL penicillin, 100 μg/mL streptomycin, and 3 µg/mL puromycin at 5% CO_2_ and 37 °C. Human coronavirus-organ culture 43 [HCoV-OC43 (ATCC; Cat. No. VR-1558)] was cultured in 293 T cells. Severe acute respiratory syndrome-coronavirus-2 (SARS-CoV-2/Hu/DP/Kng/19–020, GenBank Accession No. LC528232) was cultured in Vero cells. HCoV-OC43 titration was performed with a focus-forming assay, as previously described^28^, using an antibody against the N protein (anti-OC43, clone 541-8F (Sigma-Aldrich)). SARS-CoV-2 titrations were performed using the 50% tissue culture infectious dose (TCID50) assay. Briefly, the culture supernatants were serially diluted in Dulbecco’s Modified Eagle’s medium supplemented with 2% fetal bovine serum, 100 U/mL penicillin, and 100 μg/mL streptomycin. Confluent Vero cells in 96-well plates were infected with 50 µL of the diluted samples and incubated at 37 °C for 6 d. The cells were then fixed with 10% formalin and stained with crystal violet, and TCID_50_ was calculated using the Behrens–Karber method. Staurosporine (Sigma-Aldrich, Item No. AG-CN2-0022-C100) was dissolved in dimethyl sulfoxide. To induce apoptosis, cells were cultured in a growth medium (DMEM supplemented with 10% FBS) containing 1 µM staurosporine. For the infection study, viruses were inoculated at the indicated multiplicity of infection. The plasmids used in this study are listed in the Supplementary Table. Transient transfection of the plasmids was performed using polyethylenimine as described previously^28^.

### Measurement of luciferase activity

The NanoLuc and firefly luciferase activities were measured using the Nano-Glo Luciferase Assay System (Promega, Cat. No. N1120) and Bright-Glo Luciferase Assay System (Promega, Cat. No. E2620). The cells were washed with phosphate-buffered saline (PBS) and lysed in lysis buffer (1% Triton-X100, 50 mM Tris–HCl [pH 7.4], 150 mM NaCl, and protease inhibitors (Roche, Cat. No. 11873580001). The clear lysate was mixed with the luciferase assay buffer. The luminescence intensity of the mixture was measured using a luminometer (Varioskan LUX, Thermo Fisher Scientific) for 1 s. The values are represented as relative light units.

### Western blotting

The cells were washed with ice-cold PBS, scraped, collected by centrifugation at 500 × *g* and 4 °C for 10 min, and lysed in a lysis buffer. The lysates were mixed with 2 × Laemmli sample buffer and subjected to sodium dodecyl sulfate–polyacrylamide gel electrophoresis. The resolved proteins were transferred to an Immobilon-P polyvinylidene difluoride membrane (Millipore, Cat. No. IPVH00010). The membrane was blocked with a blocking buffer (3% or 0.3% skim milk in TBS-T [25 mM Tris (pH 7.5), 137 mM NaCl, 0.27 mM KCl, and 0.05% Tween20]) for 30 min and incubated with the primary antibodies (anti-Myc, clone 9E10, anti-α-Tubulin, clone DM1A (Sigma-Aldrich)) at 4 °C overnight. The membranes were then incubated with secondary antibodies at 25 °C for 50 min. Immunoreactive signals were developed using EzWestLumi plus (ATTO Corporation, Code No. 2332638) and observed using an iBright Imaging System (Thermo Fisher Scientific).

### Flow cytometry

293T cells were stained with propidium iodide and annexin V-FITC using the Annexin V FITC Kit (Beckman Coulter, Cat. No. IM2375). The stained cells were analyzed using CytoFLEX S (Beckman Coulter).

### Statistical analysis

The means between the two groups were compared using Student’s *t*-test. Differences were considered significant at *P < 0.01 and **P < 0.005. Additional methods and extended data displays are discussed in detail in Supplementary Information.

## Results

### Establishment of a highly sensitive FlipNanoLuc protease reporter system

**Figure 1:**
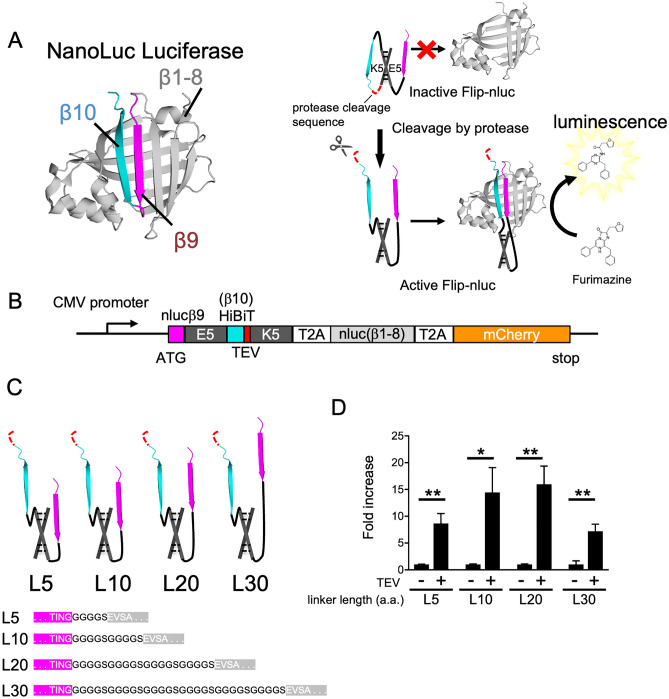
Development of NanoLuc luciferase-based protease indicator, FlipNanoLuc. (**A**) Structure of NanoLuc (left) and outline of FlipNanoLuc system. NanoLuc β-strand 1–8 (β1–8), β9, and β10 were indicated in light grey, pink, and light blue, respectively (left). NanoLuc was split into three fragments, β1–9, β9, and β10. Protease recognition sequence (red) and coiled dimerization sequence E5 and K5 (black) are flanked with β9 and β10, and β9 and β10 are locked in parallel, resulting in the prevention of self-assembly. When the sequence was cleaved by protease, β9 and β10 were anti-parallel, resulting in self-assembly and luminescence activity (right). (**B**) Primary structure of Tobacco Etch Virus protease (TEVpro)-FlipNanoLuc biosensor. Open reading frame (ORF) of NanoLuc β9: nluc9 (pink), coiled-coil dimerization sequence E5 (black), HiBiT (β10) peptides (light blue), TEVpro recognition sequence: TEV (red), coiled-coil dimerization sequence K5: K5 (black), T2A self-cleaving peptides (white), NanoLuc β1-8: nluc β1-8 (light grey), mCherry (orange), an internal control, are indicated. (**C**) Schematic structure of different lengths of linker between β9 and E5 in B. (**D**) Effect of linker length between nluc9 and E5 on FlipNanoLuc activity. FlipNanoLuc expressing vectors containing indicated linker length and either empty or TEV expressing vector were co-transfected to 293 T cells. After 48 h post-transfection, the cells were lysed and NanoLuc luciferase activity and mCherry fluorescence measured. Nanoluc activity was compensated with mCherry fluorescence. **P < 0.005, *P < 0.01 (Student’s *t*-test).

To design a NanoLuc-based luminogenic protease reporter, we used the Flip-GFP protease reporter system, which was reported previously by Zhang Q et al.^8^. It is a modified split-GFP system that can be assembled and activated by site-specific cleavage. Similar to GFP, NanoLuc has a β barrel structure at the core, and this structure can also be split into three pieces: nLuc (β1–8), and two β‐strand fragments—nLuc (β9) and nLuc (β10). It has been shown that when β9 and β10 are close, these fragments rapidly bind to β1–8, and NanoLuc assembles and achieves the catalytic activity of luciferase. This mechanism has been used to detect protein interactions of antigen-dependent variable region fragments, V_H_ and V_L_^29^. As demonstrated in the Flip-GFP system, we connected two small β strands, nLuc (β9) and nLuc (β10), with designed heterodimerizing coil linkers, E5 and K5, consisting charged residues that occupy positions e and g of the heptad repeat^30^. In this study, HiBiT was used instead of nLuc (β10). The HiBiT (β10) sequence was first described by Dixon et al.^27^, who identified 16 beneficial amino acid substitutions that increased the stability and activity of split NanoLuc luciferase in a random mutagenesis study. This variant is named NanoBiT, and the fragments are called HiBiT and LgBiT; the variant is widely used in protein detection systems. The E5/K5 linker connection creates parallel β strands by "flipping" HiBiT (β10). The E5/K5 heterodimer prevents the formation of anti-parallel β strands and self-assembly of the split NanoLuc. The protease cleavage sequence was inserted between HiBiT (β10) and K5 in the reporter-expressing cells. Owing to proteolytic cleavage, HiBiT (β10) broke down, and its configuration altered by flipping back and forming an anti-parallel structure with β9, which enabled self-assembly with β1–8. This reconstruction led to luciferase enzymatic activity and facilitated the detection of protease activity by the addition of a luminescence substrate (Fig. 1A). This reporter was named FlipNanoLuc. To prove this concept, we designed a tobacco etch virus (TEV) protease reporter by introducing a TEV cleavage sequence into FlipNanoLuc. The inserted vector contained an open reading frame (ORF) of β9-E5- β10-K5, β1-8, and mCherry, an internal marker connected to the T2A sequence, and was expressed in 293 T cells (Fig. 1B). To fine-tune the assembly of cleaved β9-E5– β10 and β1-8, we designed and tested several linker lengths between β9 and E5. As shown in Fig. 1C,D, all cells that were co-transfected with the reporter vector and TEV expression vector exhibited luminescence when the cell lysate was mixed with the NanoLuc substrate. These results indicated that β9-E5- β10 and β1–8 were assembled in a TEV cleavage-dependent manner, as expected. Among the FlipNanoLuc variant-expressing 293 T cells, FlipNanoLuc with a 20aa linker showed the largest luminescence increase (15-fold) upon TEV cleavage, suggesting that 20aa is the best linker length for efficient assembly of NanoLuc. The construct with a 20aa linker was chosen for the rest of the experiments.

### Improvement of the sensitivity and signal-to-noise ratio of FlipNanoLuc reporter system

**Figure 2:**
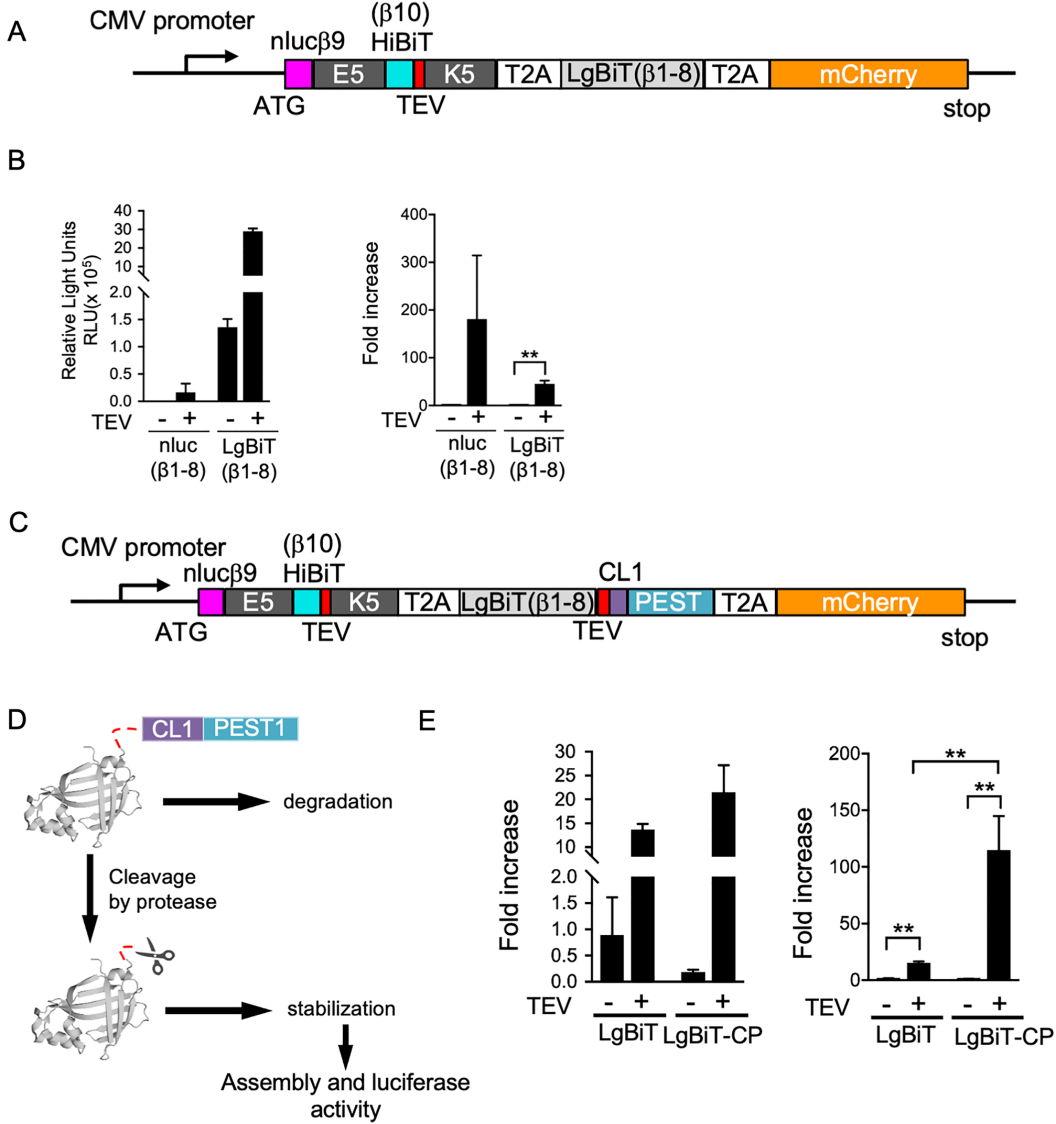
FlipNanoLuc sensitivity is increased by replacing nluc (β 1–8) as LgBiT (β 1–8) and fusing CL1-PEST1 degradation signal peptide. (**A**) Primary structure of TEVpro-FlipNanoLuc (LgBiT) biosensor. (**B**) TEV protease-dependent TEVpro-FlipNanoLuc activity in LgBiT (β1-8)-based or nLuc (β1-8)-based system. TEVpro-FlipNanoLuc expressing vector and either empty vector or TEV protease expressing vector were transfected into 293 T cells. After 48 h post-transfection, the cells lysed and measured NanoLuc luciferase activity and mCherry fluorescence. The value of FlipNanoLuc activities after compensation with mCherry fluorescence (left) and fold increase by the co-expression of TEVpro (right) are indicated. **P < 0.005 (Student’s *t-*test). (**C**) Schematic structure of TEVpro-FlipNanoLuc biosensor containing LgBiT connected with CL1-PEST1 (CP) sequence via TEV protease recognition sequence. (**D**) The principle of the reduction of background level by the addition of CP sequence to the LgBiT. (**E**) TEV protease dependent TEVpro-FlipNanoLuc activity in LgBiT-CP adopted system. After 48 h post-transfection, the cells lysed and measured NanoLuc luciferase activity and mCherry fluorescence. The value of FlipNanoLuc activities after compensation with mCherry fluorescence (left) and fold increase by the co-expression of TEVpro (right) are indicated. **P < 0.005 (Student’s *t-*test).

To improve the sensitivity, we further introduced the NanoBiT technology to the FlipNanoLuc system. We substituted LgBiT (β1–8) for NanoLuc (β1–8) (Fig. 2A) and tested TEV protease-dependent NanoLuc activity. As shown in Fig. 2B, NanoLuc activity was significantly (175-fold) increased in LgBiT (β1–8) than in conventional *O. gracilirostris*-derived NanoLuc (β1–8) (compare lanes 2 and 4 in Fig. 2B, left graph). However, the background level corresponding to NanoLuc activity in the absence of TEV protease was also increased in the LgBiT (β1–8) system, resulting in a lower increase than that in conventional NanoLuc (β1–8) (compare lanes 2 and 4 in Fig. 2B right graph). To solve this problem, we connected the rapid degradation peptide tags CL1^31,32^ and PEST1^33^ at the C-terminus of LgBiT (β1–8) through the protease cleavage sequence (Fig. 2C,D). Consequently, NanoLuc activity in the absence of TEV protease was significantly decreased even in the presence of the LgBiT (β1–8) system (Fig. 2E), and increased the signal-to-noise ratio of FlipNanoLuc activity.

### Evaluation of FlipNanoLuc system for the caspase-3 activity reporter upon apoptosis induction

**Figure 3:**
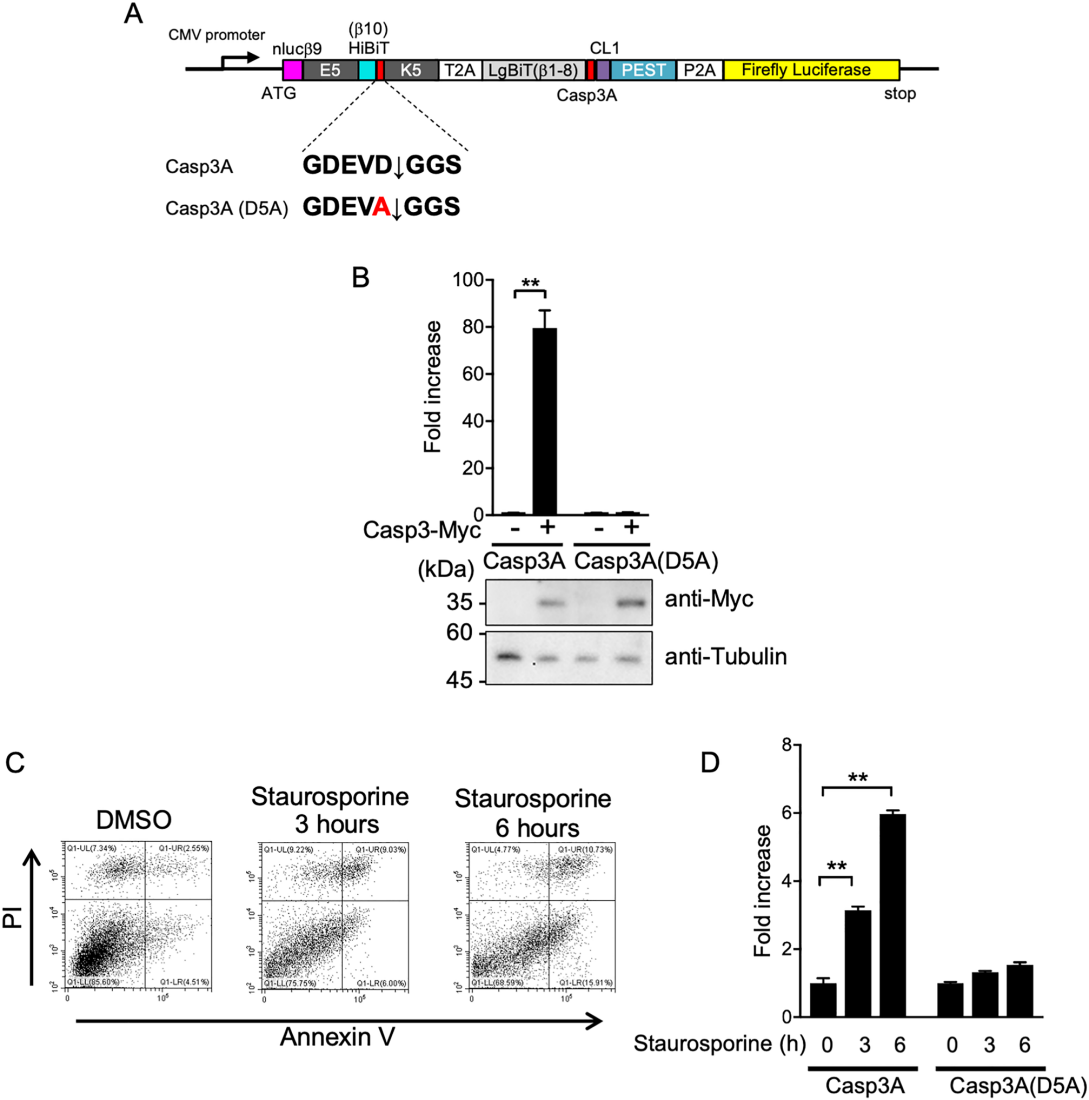
Detection of caspase-3 activity by FlipNanoLuc system in the apoptosis-induced cells. (**A**) Schematic structure of caspase-3 (Casp3A)-FlipNanoLuc protease biosensor. ORFs of Firefly luciferase (yellow) are connected via P2A self-cleaving peptides (white) at C-terminal of this construct. (**B**) Caspase-3 protease recognition sequence dependent Casp3A-FlipNanoLuc activities. Expressing vectors of Casp3A-FlipNanoLuc containing Casp3A (WT) or Casp3A (D5A), and either empty vector or Casp3A protease expressing vector were transfected into 293 T cells. After 24 h post-transfection, the cells were lysed and NanoLuc and Firefly luciferase activities were measured. Fold increase of the value of FlipNanoLuc activities after compensation with Firefly luciferase activities are indicated. **P < 0.005 (Student’s *t*-test). The expression levels of Myc-tagged caspase-3 protease and endogenous α-Tubulin were confirmed by western blotting (bottom panels). (**C**) Flow cytometry analysis of apoptotic 293 T cells stably expressing biosensor by staining with propidium iodide (PI) and Annexin V-FITC. After 0, 3, 6 h treatment with 1 µM staurosporin, 293 T cells were stained with PI/Annexin V-FITC and then analyzed by flow cytometry. (**D**) Casp3A-FlipNanoLuc activity in Staurosporine treated cells. Casp3A-FlipNanoLuc or Casp3A(D5A)-FlipNanoLuc expressing 293 T cells were treated with 1 µM Staurosporine for an indicated time. The cells were lysed and NanoLuc luciferase and Firefly luciferase activities were measured. Fold increase of the value of FlipNanoLuc activities after compensation with Firefly luciferase activities are indicated. n.s.: not significant, **P < 0.005 (Student’s *t-*test).

First, we evaluated the FlipNanoLuc protease biosensor as an indicator of caspase activity. Caspase-3 is a frequently activated endoprotease that regulates the inflammation and apoptosis signaling networks^34^. Caspase-3 is a cysteine protease activator of caspase-6 and caspase-2 and metabolizes the amyloid β/A4 precursor protein^35–37^. Its chromogenic and fluorogenic peptide substrates have been used to determine caspase activity in apoptotic cells^38^. Caspase-3 recognition sequence was introduced into the FlipNanoLuc system (Fig. 3A) and tested for NanoLuc activity upon caspase-3 overexpression. NanoLuc activity was increased 80-fold in cells co-transfected with caspase-3 expression vector compared with that of cells co-transfected with empty vector. However, this increase was not detected in FlipNanoLuc-expressing cells that have single amino acid substitution in aspartic acid residue at the caspase-3 recognition sequence, Casp3A (D5A), although sufficient amount of caspase-3 was expressed (Fig. 3B). These results indicate that this FlipNanoLuc caspase-3 biosensor was specifically activated by exogenously expressed caspase-3. Next, this biosensor was tested for NanoLuc activity upon apoptosis induction. To minimize the effect of biosensor expression on the apoptosis induction, stably expressing cells were generated using a retroviral transduction system. Staurosporine is a relatively non-selective protein kinase inhibitor that blocks many kinases. Staurosporine is often used for inducing Caspase-3-mediated apoptosis^39,40^. As shown in Fig. 3C,D, there was over a sixfold gradual increase in NanoLuc activity after staurosporine treatment, although this increase was not observed in Casp3A (D5A) FlipNanoLuc-expressing cells even when apoptosis was induced. These results indicate that the Caspase-3 FlipNanoLuc protease reporter system functions as an indicator of apoptosis signaling.

### Evaluation for the coronavirus protease reporter system

**Figure 4:**
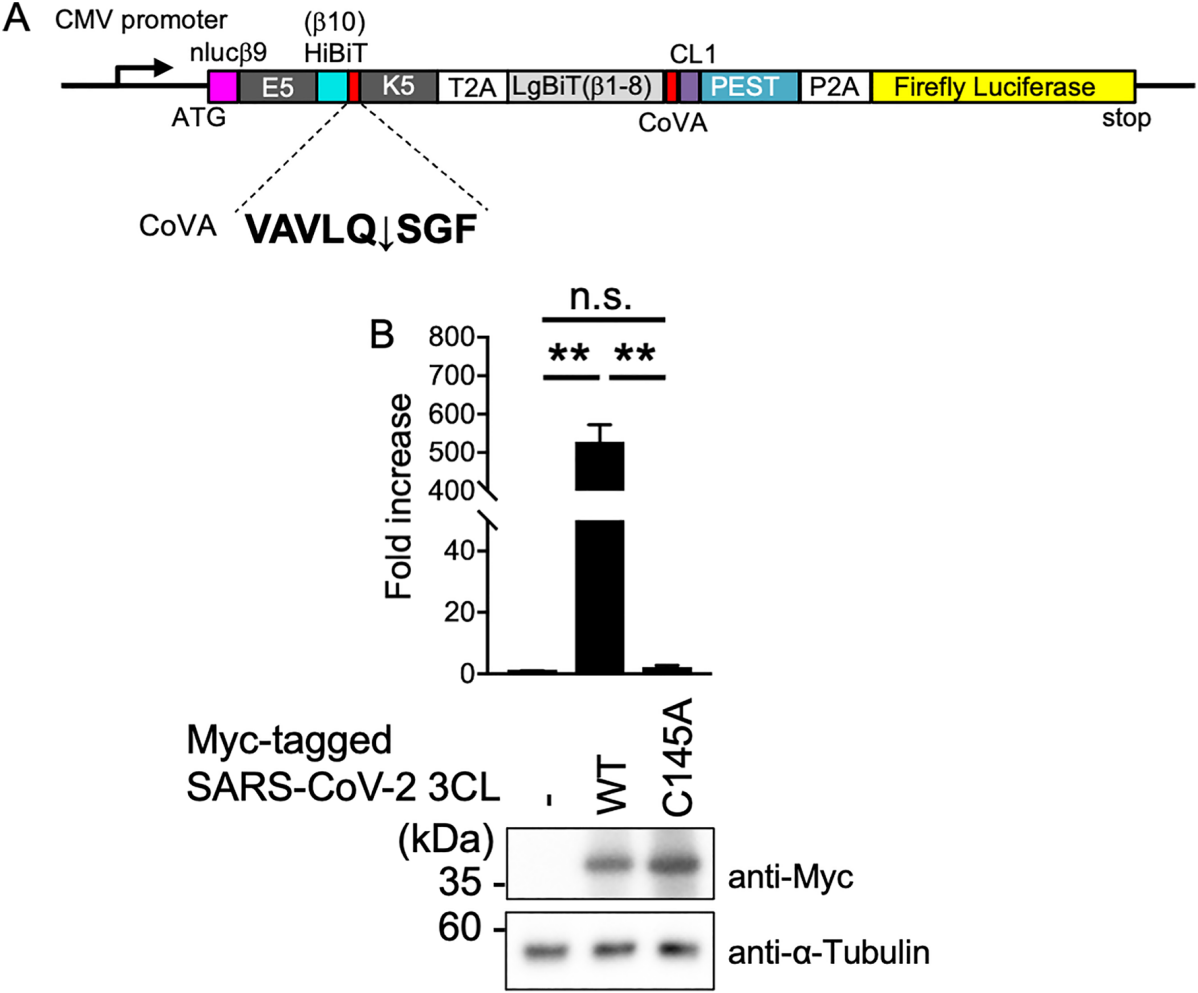
Detection of betacoronavirus 3CL protease activity by the FlipNanoLuc system. (**A**) Schematic structure of 3CL protease (3CLpro)-FlipNanoLuc protease biosensor. (**B**) SARS-CoV-2 3CL protease-dependent 3CLpro-FlipNanoLuc activities. 3CLpro-FlipNanoLuc expressing vector and either empty vector, wild-type 3CL protease, or C145A mutant of 3CL protease expressing vector were transfected into 293 T cells. After 48 h post-transfection, the cells were lysed and NanoLuc and Firefly luciferase activities were measured. Fold increase of the value of FlipNanoLuc activities after compensation with Firefly luciferase activities are indicated. n.s.: not significant, **P < 0.005 (Student’s *t-*test). The expression levels of Myc-tagged SARS-CoV-2 3CL protease and endogenous α-Tubulin were confirmed by western blotting (bottom panels).

To evaluate the viability of the FlipNanoLuc system as a reporter that can detect the activities of viral proteases, we introduced the coronavirus 3CLpro recognition sequence into this system. Cells expressing this reporter can also be utilized as a cell indicator to detect coronavirus infection under non-invasive and non-staining conditions. Coronaviruses produce two large viral polyproteins: ORF1a and ORF1b. These proteins are processed by two cysteine proteases, 3CLpro and PLpro, which are encoded by the viral genome. The main protease is 3CLpro, and processing by this enzyme is essential for viral replication. 3CLpro processes viral ORF1a and ORF1b at more than 10 junctions to generate non-structural proteins such as RdRp, helicase, and 3CLpro itself^1^. By introducing the 3CLpro recognition consensus sequence (VAVLQSGF)^41^ into the FlipNanoLuc system (Fig. 4A), the simultaneous expression of the wild-type (WT) (but not the catalytically inactive mutant (C145A)) of SARS-CoV-2 3CLpro significantly increased NanoLuc activity (Fig. 4B).

**Figure 5:**
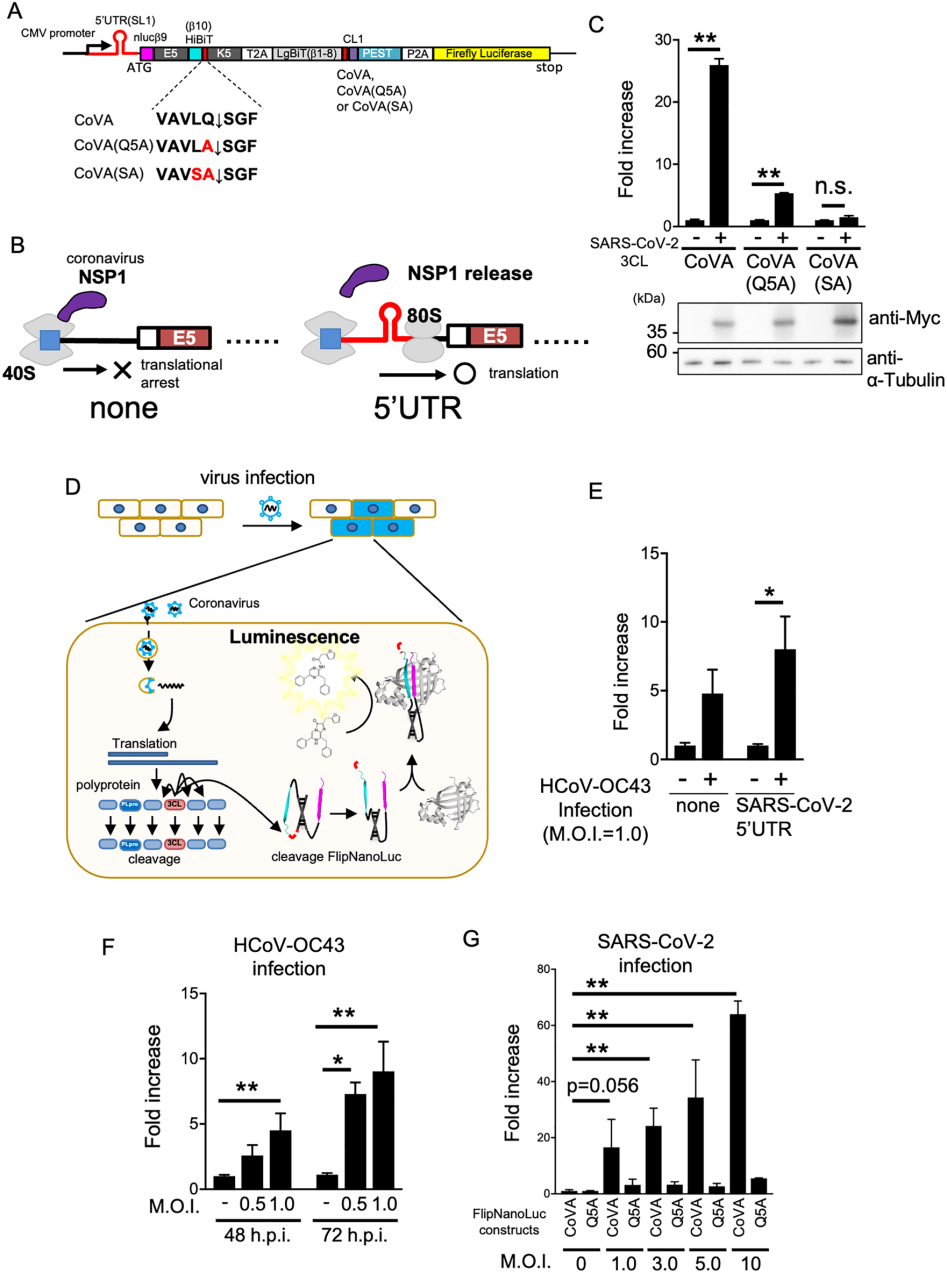
Detection of betacoronavirus infections by FlipNanoLuc system. (**A**) Primary structure of 3CLpro-FlipNanoLuc protease biosensor containing SARS-CoV-2 derived stem loop 1 (SL1) sequence in its mRNA. SARS-CoV-2 SL1 (red line) are added at 5' end of ORF coding sequence. Mutated amino acid in protease recognition sequence are indicated as red characters. (**B**) Schematic presentation indicating the coronavirus NSP1 mediated global translational arrest (left) and escaping by Coronavirus 5′′UTR. (**C**) SARS-CoV-2 3CL protease recognition sequence dependent 3CLpro-FlipNanoLuc activities. Expressing vectors of 3CLpro-FlipNanoLuc containing CoVA (WT), CoVA (Q5A) or CoVA (SA), and either empty vector or wild-type 3CL protease expressing vector were transfected into 293 T cells. After 48 h post-transfection, the cells were lysed and NanoLuc and Firefly luciferase activities were measured. Fold increase of the value of FlipNanoLuc activities after compensation with Firefly luciferase activities are indicated. n.s.: not significant, **P < 0.005 (Student’s *t*-test). The expression levels of Myc-tagged SARS-CoV-2 3CL protease and endogenous α-Tubulin were confirmed by western blotting (bottom panels). (**D**) Schematic presentation of the detection of coronavirus infection by FlipNanoLuc system. The infection of coronavirus induces the expression of 3CL protease for processing their own polyproteins. If the cells are expressing 3CLpro-FlipNanoLuc construct, viral 3CL protease simultaneously cleaves biosensor, results in the formation of NanoLuc luciferase. (**E**) 3CLpro-FlipNanoLuc activities in cells infected with human coronavirus strain OC43 (HCoV-OC43) were increased by the addition of stem loop 1 (SL1). 293 T cells were transfected with 3CLpro-FlipNanoLuc or 3CLpro-SL1-FlipNanoLuc expressing vector. After 24 h post-transfection, the cells were infected with HCoV-OC43 at MOI = 1.0. After 48 h post-infection, the cells were lysed and NanoLuc and Firefly luciferase activities were measured. Fold increase of the value of FlipNanoLuc activities after compensation with Firefly luciferase activities are indicated. n.s.: not significant, **: *P* < 0.005 (Student’s t-test). (**F**) M.O.I. dependent increase of 3CLpro-SL1-FlipNanoLuc activities in HCoV-OC43 infected cells. The 293 T cells stably expressing biosensors were infected with HCoV-OC43 at indicated M.O.I., then cells were harvested at 48 h or 72 h post-infection. Fold increase of the value of FlipNanoLuc activities are indicated. n.s.: not significant, **P < 0.005 (Student’s t-test). (**G**) 3CLpro-SL1-FlipNanoLuc biosensor detect SARS-CoV-2 infection. After 24 h post-transfection, the BHK-hACE2 cells were infected with SARS-CoV-2 at indicated M.O.I. and incubated for 48 h. Fold increase of the value of FlipNanoLuc activities are indicated. n.s.: not significant, **P < 0.005 (Student’s *t*^-^test).

Subsequently, we tested whether this FlipNanoLuc-based 3CL protease reporter could be used in viral infection detection systems. There was a problem that the expression level of reporter gene was significantly reduced only in coronavirus-infected cells, probably due to the coronavirus NSP-mediated global translation shutdown of the host cells. Although cellular protein synthesis is impaired by viral NSP1 binding to the 40S subunit in ribosomal complexes, viral protein synthesis is not impaired by the 5ʹ untranslated region (5ʹUTR) of viral mRNA (Fig. 5A)^42^. This shutdown/escape system facilitates the translation of viral proteins in infected cells. Since the stem-loop 1 (SL1) structure in the 5ʹ UTR responds to escape from NSP1-mediated shutdown, we introduced the SL1 sequence into the 5ʹ end of the 3CLpro-FlipNanoLuc system (Fig. 5B).

Furthermore, we constructed a negative control of 3CLpro-FlipNanoLuc by substituting the amino acid alanine at the fifth position for glutamine or additionally substituting serine at the fourth position for leucine in the protease recognition sequence (Fig. 5A). We confirmed that these mutations significantly reduced NanoLuc activity on 3CLpro, suggesting that the NanoLuc activity induced by the co-expression of 3CLpro was dependent on the recognition sequence (Fig. 5C).

Finally, we tested whether the 3CLpro-FlipNanoLuc system could detect the coronavirus infection. In cells infected with coronaviruses, 3CL protease is expressed to cleave its own polyproteins, ORF1a and ORF1b. When 3CLpro-FlipNanoLuc was expressed in these cells, 3CL protease cleaved it simultaneously, resulting in NanoLuc formation that only occurred in the virus-infected cells (Fig. 5D). We tested the viability of the system using the human coronavirus strain OC43 (HCoV-OC43). As shown in Fig. 5E , 3CLpro -FlipNanoLuc activity increased in HCoV-OC43-infected cells compared with that in mock-infected cells. Furthermore, the addition of SL1 significantly enhanced these signals. These signals were multiplicity of infection (MOI)- and time-dependent (Fig. 5F). The same experiment was performed using SARS-CoV-2. As shown in Fig. 5G, 3CLpro-FlipNanoLuc activity was significantly increased in cells infected with SARS-CoV-2, and this increase was MOI-dependent.

## Discussion

In this study, we successfully established a novel protease reporter system based on highly sensitive NanoLuc luciferase. Furthermore, we demonstrated that this system can be used for the detection of coronavirus infection. The plaque or TCID50 assay is generally used to titrate viral infectivity. However, these traditional methods take a week to yield results. In contrast, the FlipNanoLuc system enables results to be obtained within an hour. Furthermore, it requires only simple and easy handling, indicating a great advantage in high-throughput screening of antiviral reagents or isolation of clinical samples.

In this study, we detected both HCoV-OC43 and SARS-CoV-2 infections using the same protease cleavage sequence. Protease cleavage sites are conserved in the *Coronaviridae* family. In addition to HCoV-OC43 and SARS-CoV-2, a previous report indicated that the amino acid sequence used in this study is also recognized by HCoV-NL63, which belongs to alphacoronaviruses, and infectious bronchitis virus, which belongs to gammacoronaviruses^41^. These results suggest that this reporter system may also be a feasible method for detecting novel coronaviruses that may emerge in the future. However, substrate specificity has been reported^41^. The substitution of Gln at position -1 to His or Met or Leu at position -2 to Cys or Met retained the susceptibility to HCoV-OC43 3CL protease, whereas these substitutions altered the relative resistance to SARS-CoV-1 3CL protease. These results suggest that the introduction of these mutations in FlipNanoLuc facilitates the development of OC43 specific biosensor.

To date, three independent groups have screened small compound inhibitors for the SARS-CoV-2 3CL protease using the FlipGFP-based protease reporter system^25,26,43^. However, all of them focused on the protease activities of the overexpressed 3CL proteins, but none demonstrated the detection of the viral infection itself using this system. We also tested whether the FlipGFP-based system could detect coronavirus infection; however, no positive results were obtained (Supplementary Fig. S2). The results of this study suggest that the sensitivity of FlipNanoLuc is much higher than that of FlipGFP. Although the FlipGFP system has been utilized for the detection of apoptosis in zebrafish embryos and *Drosophila* midgut^8^, our FlipNanoLuc system is a promising tool for detecting apoptosis with a higher sensitivity. We believe that this system has the potential to be used as an indicator of endogenous proteases with very low expression levels, such as trypsin, metalloproteinases, and other pathogen proteases.

## Supplementary Information

Supplementary Information 1.

Supplementary Information 2.

## Data Availability

The data supporting the findings of this study are available from the corresponding author, Eiji Morita, upon reasonable request.

## Abbreviations

SARS-CoV-2: Severe acute respiratory syndrome-coronavirus-2
3CL: 3C-like
OC43: Organ culture 43
CAD: Caspase-activated DNase

## References

[1] V’kovski, P., Kratzel, A., Steiner, S., Stalder, H. & Thiel, V. Coronavirus biology and replication: implications for SARS-CoV-2. *Nat. Rev. Microbiol.* **19**, 155–170 (2021). 10.1038/s41579-020-00468-6 PMC7592455 33116300

[2] Hong M-L, Li L-J, Han H-X, Chu X (2014). A label-free fluorescence assay for trypsin based on the electron transfer between oligonucleotide-stabilized Ag nanoclusters and cytochrome c. Anal. Sci..

[3] Poon C-Y (2016). FRET-based modified graphene quantum dots for direct trypsin quantification in urine. Anal. Chim. Acta.

[4] Işık B, Sezgintürk MK (2017). Quantification of trypsin activity by a new biosensing system based on the enzymatic degradation and the destructive nature of trypsin. Int. J. Pept. Res. Ther..

[5] Ong ILH, Yang K-L (2017). Recent developments in protease activity assays and sensors. Analyst.

[6] Nicholls SB, Chu J, Abbruzzese G, Tremblay KD, Hardy JA (2011). Mechanism of a genetically encoded dark-to-bright reporter for caspase activity. J. Biol. Chem..

[7] Ding Y (2015). Ratiometric biosensors based on dimerization-dependent fluorescent protein exchange. Nat. Methods.

[8] Zhang Q (2019). Designing a green fluorogenic protease reporter by flipping a beta strand of gfp for imaging apoptosis in animals. J. Am. Chem. Soc..

[9] To T-L (2016). Rational design of a GFP-based fluorogenic caspase reporter for imaging apoptosis in vivo. Cell Chem. Biol..

[10] Nicholls SB, Hardy JA (2013). Structural basis of fluorescence quenching in caspase activatable-GFP. Protein Sci..

[11] Tian F (2021). Noninvasive bioluminescence imaging of matrix metalloproteinase-14 activity in lung cancer using a membrane-bound biosensor. Anal. Chem..

[12] Swisher LZ (2014). Quantitative electrochemical detection of cathepsin B activity in complex tissue lysates using enhanced AC voltammetry at carbon nanofiber nanoelectrode arrays. Biosens. Bioelectron..

[13] Song Y (2019). electrochemical activity assay for protease analysis using carbon nanofiber Nanoelectrode arrays. Anal. Chem..

[14] Wang Q, Yu L, Wong RCH, Lo P-C (2019). Construction of cathepsin B-responsive fluorescent probe and photosensitizer using a ferrocenyl boron dipyrromethene dark quencher. Eur. J. Med. Chem..

[15] Amouzadeh Tabrizi M, Ferré-Borrull J, Marsal LF (2020). An optical biosensor for the determination of cathepsin B as a cancer-associated enzyme using nanoporous anodic alumina modified with human serum albumin-thionine. Mikrochim. Acta.

[16] Zhou J (2017). Assessing activity of Hepatitis A virus 3C protease using a cyclized luciferase-based biosensor. Biochem. Biophys. Res. Commun..

[17] Hsieh M-S (2017). Detection and quantification of dengue virus using a novel biosensor system based on dengue NS3 protease activity. PLoS ONE.

[18] Wang L (2010). Bioluminescence imaging of Hepatitis C virus NS3/4A serine protease activity in cells and living animals. Antiviral Res..

[19] Hu K (2005). A human immunodeficiency virus type 1 protease biosensor assay using bioluminescence resonance energy transfer. J. Virol. Methods.

[20] Oka T (2011). Bioluminescence technologies to detect calicivirus protease activity in cell-free system and in infected cells. Antiviral Res..

[21] Mathieu C (2021). A Bioluminescent 3CLPro Activity Assay to Monitor SARS-CoV-2 Replication and Identify Inhibitors. Viruses.

[22] Gerber PP (2022). A protease-activatable luminescent biosensor and reporter cell line for authentic SARS-CoV-2 infection. PLOS Pathog..

[23] Rawson JMO, Duchon A, Nikolaitchik OA, Pathak VK, Hu W-S (2021). Development of a cell-based luciferase complementation assay for identification of SARS-CoV-2 3CLpro inhibitors. Viruses.

[24] O’Brien A (2021). Detecting SARS-CoV-2 3CLpro expression and activity using a polyclonal antiserum and a luciferase-based biosensor. Virology.

[25] Ma C (2021). Discovery of SARS-CoV-2 papain-like protease inhibitors through a combination of high-throughput screening and a FlipGFP-based reporter assay. ACS Cent. Sci..

[26] Xia Z (2021). Rational design of hybrid SARS-CoV-2 main protease inhibitors guided by the superimposed cocrystal structures with the peptidomimetic inhibitors GC-376, Telaprevir, and Boceprevir. ACS Pharmacol. Transl. Sci..

[27] Dixon AS (2016). NanoLuc complementation reporter optimized for accurate measurement of protein interactions in cells. ACS Chem. Biol..

[28] Tabata K (2016). Unique requirement for ESCRT factors in flavivirus particle formation on the endoplasmic reticulum. Cell Rep..

[29] Ohmuro-Matsuyama Y, Ueda H (2018). Homogeneous noncompetitive luminescent immunodetection of small molecules by ternary protein fragment complementation. Anal. Chem..

[30] De Crescenzo G, Litowski JR, Hodges RS, O’Connor-McCourt MD (2003). Real-time monitoring of the interactions of two-stranded de novo designed coiled-coils: Effect of chain length on the kinetic and thermodynamic constants of binding. Biochemistry.

[31] Gilon T, Chomsky O, Kulka RG (1998). Degradation signals for ubiquitin system proteolysis in Saccharomyces cerevisiae. EMBO J..

[32] Bence NF, Sampat RM, Kopito RR (2001). Impairment of the ubiquitin-proteasome system by protein aggregation. Science.

[33] Ghoda L, Sidney D, Macrae M, Coffino P (1992). Structural elements of ornithine decarboxylase required for intracellular degradation and polyamine-dependent regulation. Mol. Cell. Biol..

[34] Green DR, Llambi F (2015). Cell death signaling. Cold Spring Harb. Perspect. Biol..

[35] Slee EA (1999). Ordering the cytochrome c-initiated caspase cascade: hierarchical activation of caspases-2, -3, -6, -7, -8, and -10 in a caspase-9-dependent manner. J. Cell Biol..

[36] Gervais FG (1999). Involvement of caspases in proteolytic cleavage of Alzheimer’s amyloid-beta precursor protein and amyloidogenic A beta peptide formation. Cell.

[37] Tesco G, Koh YH, Tanzi RE (2003). Caspase activation increases beta-amyloid generation independently of caspase cleavage of the beta-amyloid precursor protein (APP). J. Biol. Chem..

[38] Lei Q (2022). Biosensors for Caspase-3: From chemical methodologies to biomedical applications. Talanta.

[39] Chae HJ (2000). Molecular mechanism of staurosporine-induced apoptosis in osteoblasts. Pharmacol. Res..

[40] Tee AR, Proud CG (2001). Staurosporine inhibits phosphorylation of translational regulators linked to mTOR. Cell Death Differ..

[41] Chuck C-P, Chow H-F, Wan DC-C, Wong K-B (2011). Profiling of substrate specificities of 3C-like proteases from group 1, 2a, 2b, and 3 Coronaviruses. PLoS ONE.

[42] Banerjee AK (2020). SARS-CoV-2 disrupts splicing, translation, and protein trafficking to suppress host defenses. Cell.

[43] Froggatt, H. M., Heaton, B. E. & Heaton, N. S. Development of a Fluorescence-Based, High-Throughput SARS-CoV-2 3CL pro Reporter Assay. *J. Virol.* **94**, (2020). 10.1128/JVI.01265-20 PMC7592234 32843534

