# Supplementary material for: A highly sensitive NanoLuc-based protease biosensor for detecting apoptosis and SARS-CoV-2 infection
Source: Sci Rep. 2023 Jan 31;13:1753. doi: 10.1038/s41598-023-28984-4 (PMC9887574; doi:10.1038/s41598-023-28984-4)
Supplement: Supplementary file 1 — Supplementary Information 1. [file 41598_2023_28984_MOESM1_ESM.docx]

**Supplementary Information: A highly sensitive NanoLuc-based protease biosensor for detecting apoptosis and SARS-CoV-2 infection**

Masashi Arakawa1,2, Akiho Yoshida3,5, Shinya Okamura3,5, Hirotaka Ebina3,4,5, Eiji Morita1,2 *

1Department of Biochemistry and Molecular Biology, Faculty of Agriculture and Life Science, Hirosaki University, Japan

2Division of Biomolecular Function, Bioresources Science, United Graduate School of Agricultural Sciences, Iwate University, Morioka, 020-0066, Japan

3Virus Vaccine Group, BIKEN Innovative Vaccine Research Alliance Laboratories, Institute for Open and Transdisciplinary Research Initiatives, Suita, Osaka University, Osaka, Japan

4Virus Vaccine Group, BIKEN Innovative Vaccine Research Alliance Laboratories, Research Institute for Microbial Diseases, Osaka University, Suita, Osaka, Japan.

5The Research Foundation for Microbial Diseases of Osaka University, Suita, Osaka, Japan

Running title: Highly sensitive NanoLuc-based protease biosensor

*Corresponding Author: Eiji Morita

Department of Biochemistry and Molecular Biology

Faculty of Agriculture and Life Science

Hirosaki University, 3 Bunkyo-cho, Hirosaki-shi, Aomori 036-8561, Japan

Phone: + 81 172 39 3586

E-mail: moritae@hirosaki-u.ac.jp

Supplementary Tables

Supplementary Figures S1-S2

pQC.Flip-nluc[L20]-T2A-mCh[TEV] in Figure 1B:

MGSLLFRVTINGGGGGSGGGGSGGGGSGGGGSEVSALEKEVSALEKEVSALEKEVSALEKEVSALEKEKVSGWRLFKKISGSSENLYFQSKVSALKEKVSALKEKVSALKEKVSALKEKVSALKEEFEGRGSLLTCGDVEENPGPKLATMVFTLEDFVGDWRQTAGYNLDQVLEQGGVSSLFQNLGVSVTPIQRIVLSGENGLKIDIHVIIPYEGLSGDQMGQIEKIFKVVYPVDDHHFKVILHYGTLVIDGVTPNMIDYFGRPYEGIAVFDGKKITVTGTLWNGNKIIDERLINPDGGRSREGRGSLLTCGDVEENPGPELMVSKGEEDNMAIIKEFMRFKVHMEGSVNGHEFEIEGEGEGRPYEGTQTAKLKVTKGGPLPFAWDILSPQFMYGSKAYVKHPADIPDYLKLSFPEGFKWERVMNFEDGGVVTVTQDSSLQDGEFIYKVKLRGTNFPSDGPVMQKKTMGWEASSERMYPEDGALKGEIKQRLKLKDGGHYDAEVKTTYKAKKPVQLPGAYNVNIKLDITSHNEDYTIVEQYERAEGRHSTGGMDELYK*

pQC.Flip-nluc(LgBiT1-8)[TEV] in Figure 2A:

MGSLLFRVTINGGGGGSGGGGSGGGGSGGGGSEVSALEKEVSALEKEVSALEKEVSALEKEVSALEKEKVSGWRLFKKISGSSENLYFQSKVSALKEKVSALKEKVSALKEKVSALKEKVSALKEEFEGRGSLLTCGDVEENPGPKLATMVFTLEDFVGDWEQTAAYNLDQVLEQGGVSSLLQNLAVSVTPIQRIVRSGENALKIDIHVIIPYEGLSADQMAQIEEVFKVVYPVDDHHFKVILPYGTLVIDGVTPNMLNYFGRPYEGIAVFDGKKITVTGTLWNGNKIIDERLITPDGGRSREGRGSLLTCGDVEENPGPELMVSKGEEDNMAIIKEFMRFKVHMEGSVNGHEFEIEGEGEGRPYEGTQTAKLKVTKGGPLPFAWDILSPQFMYGSKAYVKHPADIPDYLKLSFPEGFKWERVMNFEDGGVVTVTQDSSLQDGEFIYKVKLRGTNFPSDGPVMQKKTMGWEASSERMYPEDGALKGEIKQRLKLKDGGHYDAEVKTTYKAKKPVQLPGAYNVNIKLDITSHNEDYTIVEQYERAEGRHSTGGMDELYK*

pQC.Flip-nluc(LgBiT1-8)CP[TEV] in Figure 2C:

MGSLLFRVTINGGGGGSGGGGSGGGGSGGGGSEVSALEKEVSALEKEVSALEKEVSALEKEVSALEKEKVSGWRLFKKISGSSENLYFQSKVSALKEKVSALKEKVSALKEKVSALKEKVSALKEEFEGRGSLLTCGDVEENPGPKLATMVFTLEDFVGDWEQTAAYNLDQVLEQGGVSSLLQNLAVSVTPIQRIVRSGENALKIDIHVIIPYEGLSADQMAQIEEVFKVVYPVDDHHFKVILPYGTLVIDGVTPNMLNYFGRPYEGIAVFDGKKITVTGTLWNGNKIIDERLITPDGENLYFQSQLGGSEVACKNWFSSLSHFVIHLNSHGFPPEVEEQAAGTLPMSCAQESGMDRHPAACASARINVGGRSREGRGSLLTCGDVEENPGPELMVSKGEEDNMAIIKEFMRFKVHMEGSVNGHEFEIEGEGEGRPYEGTQTAKLKVTKGGPLPFAWDILSPQFMYGSKAYVKHPADIPDYLKLSFPEGFKWERVMNFEDGGVVTVTQDSSLQDGEFIYKVKLRGTNFPSDGPVMQKKTMGWEASSERMYPEDGALKGEIKQRLKLKDGGHYDAEVKTTYKAKKPVQLPGAYNVNIKLDITSHNEDYTIVEQYERAEGRHSTGGMDELYK*

pQC.Flip-nluc(LgBiT1-8)CP[Casp3A] in Figure 3A:

MGSLLFRVTINGGGGGSGGGGSGGGGSGGGGSEVSALEKEVSALEKEVSALEKEVSALEKEVSALEKEKVSGWRLFKKISGSSGDEVDGGSKVSALKEKVSALKEKVSALKEKVSALKEKVSALKEEFEGRGSLLTCGDVEENPGPKLATMVFTLEDFVGDWEQTAAYNLDQVLEQGGVSSLLQNLAVSVTPIQRIVRSGENALKIDIHVIIPYEGLSADQMAQIEEVFKVVYPVDDHHFKVILPYGTLVIDGVTPNMLNYFGRPYEGIAVFDGKKITVTGTLWNGNKIIDERLITPDGGDEVDGGSQLGGSEVACKNWFSSLSHFVIHLNSHGFPPEVEEQAAGTLPMSCAQESGMDRHPAACASARINVGGRSRATNFSLLKQAGDVEENPGPELMEDAKNIKKGPAPFYPLEDGTAGEQLHKAMKRYALVPGTIAFTDAHIEVDITYAEYFEMSVRLAEAMKRYGLNTNHRIVVCSENSLQFFMPVLGALFIGVAVAPANDIYNERELLNSMGISQPTVVFVSKKGLQKILNVQKKLPIIQKIIIMDSKTDYQGFQSMYTFVTSHLPPGFNEYDFVPESFDRDKTIALIMNSSGSTGLPKGVALPHRTACVRFSHARDPIFGNQIIPDTAILSVVPFHHGFGMFTTLGYLICGFRVVLMYRFEEELFLRSLQDYKIQSALLVPTLFSFFAKSTLIDKYDLSNLHEIASGGAPLSKEVGEAVAKRFHLPGIRQGYGLTETTSAILITPEGDDKPGAVGKVVPFFEAKVVDLDTGKTLGVNQRGELCVRGPMIMSGYVNNPEATNALIDKDGWLHSGDIAYWDEDEHFFIVDRLKSLIKYKGYQVAPAELESILLQHPNIFDAGVAGLPDDDAGELPAAVVVLEHGKTMTEKEIVDYVASQVTTAKKLRGGVVFVDEVPKGLTGKLDARKIREILIKAKKGGKIAV*

pQC.Flip-nluc(LgBiT1-8)CP[CoVA] in Figure 4A:

MGSLLFRVTINGGGGGSGGGGSGGGGSGGGGSEVSALEKEVSALEKEVSALEKEVSALEKEVSALEKEKVSGWRLFKKISGSSVAVLQSGFKVSALKEKVSALKEKVSALKEKVSALKEKVSALKEEFEGRGSLLTCGDVEENPGPKLATMVFTLEDFVGDWEQTAAYNLDQVLEQGGVSSLLQNLAVSVTPIQRIVRSGENALKIDIHVIIPYEGLSADQMAQIEEVFKVVYPVDDHHFKVILPYGTLVIDGVTPNMLNYFGRPYEGIAVFDGKKITVTGTLWNGNKIIDERLITPDGVAVLQSGFQLGGSEVACKNWFSSLSHFVIHLNSHGFPPEVEEQAAGTLPMSCAQESGMDRHPAACASARINVGGRSRATNFSLLKQAGDVEENPGPELMEDAKNIKKGPAPFYPLEDGTAGEQLHKAMKRYALVPGTIAFTDAHIEVDITYAEYFEMSVRLAEAMKRYGLNTNHRIVVCSENSLQFFMPVLGALFIGVAVAPANDIYNERELLNSMGISQPTVVFVSKKGLQKILNVQKKLPIIQKIIIMDSKTDYQGFQSMYTFVTSHLPPGFNEYDFVPESFDRDKTIALIMNSSGSTGLPKGVALPHRTACVRFSHARDPIFGNQIIPDTAILSVVPFHHGFGMFTTLGYLICGFRVVLMYRFEEELFLRSLQDYKIQSALLVPTLFSFFAKSTLIDKYDLSNLHEIASGGAPLSKEVGEAVAKRFHLPGIRQGYGLTETTSAILITPEGDDKPGAVGKVVPFFEAKVVDLDTGKTLGVNQRGELCVRGPMIMSGYVNNPEATNALIDKDGWLHSGDIAYWDEDEHFFIVDRLKSLIKYKGYQVAPAELESILLQHPNIFDAGVAGLPDDDAGELPAAVVVLEHGKTMTEKEIVDYVASQVTTAKKLRGGVVFVDEVPKGLTGKLDARKIREILIKAKKGGKIAV*

pQC.SARS2_5'UTR(SL1)-Flip-nluc(LgBiT1-8)CP[CoVA]-Fluc in Figure 5A:

attaaaggtttataccttcccaggtaacaaaccaaccaggcctcgtacgcttaattaacggccaccMGSLLFRVTINGGGGGSGGGGSGGGGSGGGGSEVSALEKEVSALEKEVSALEKEVSALEKEVSALEKEKVSGWRLFKKISGSSVAVLQSGFKVSALKEKVSALKEKVSALKEKVSALKEKVSALKEEFEGRGSLLTCGDVEENPGPKLATMVFTLEDFVGDWEQTAAYNLDQVLEQGGVSSLLQNLAVSVTPIQRIVRSGENALKIDIHVIIPYEGLSADQMAQIEEVFKVVYPVDDHHFKVILPYGTLVIDGVTPNMLNYFGRPYEGIAVFDGKKITVTGTLWNGNKIIDERLITPDGVAVLQSGFQLGGSEVACKNWFSSLSHFVIHLNSHGFPPEVEEQAAGTLPMSCAQESGMDRHPAACASARINVGGRSRATNFSLLKQAGDVEENPGPELMEDAKNIKKGPAPFYPLEDGTAGEQLHKAMKRYALVPGTIAFTDAHIEVDITYAEYFEMSVRLAEAMKRYGLNTNHRIVVCSENSLQFFMPVLGALFIGVAVAPANDIYNERELLNSMGISQPTVVFVSKKGLQKILNVQKKLPIIQKIIIMDSKTDYQGFQSMYTFVTSHLPPGFNEYDFVPESFDRDKTIALIMNSSGSTGLPKGVALPHRTACVRFSHARDPIFGNQIIPDTAILSVVPFHHGFGMFTTLGYLICGFRVVLMYRFEEELFLRSLQDYKIQSALLVPTLFSFFAKSTLIDKYDLSNLHEIASGGAPLSKEVGEAVAKRFHLPGIRQGYGLTETTSAILITPEGDDKPGAVGKVVPFFEAKVVDLDTGKTLGVNQRGELCVRGPMIMSGYVNNPEATNALIDKDGWLHSGDIAYWDEDEHFFIVDRLKSLIKYKGYQVAPAELESILLQHPNIFDAGVAGLPDDDAGELPAAVVVLEHGKTMTEKEIVDYVASQVTTAKKLRGGVVFVDEVPKGLTGKLDARKIREILIKAKKGGKIAV*

pQC.FlipGFP[CoVA] in Supplemental Figure 2A:

MDLPDDHYLSTQTILSKDLNSGLRSGSGLEMEVSALEKEVSALEKEVSALEKEVSALEKEVSALEKEKRDHMVLLEYVTAAGITGSSVAVLQSGFKVSALKEKVSALKEKVSALKEKVSALKEKVSALKEEFEGRGSLLTCGDVEENPGPKLATMRKGEELFTGIVPILVELDGDVNGHKFFVRGEGEGDATIGKLSLKFICTTGKLPVPWPTLVTTLTYGVQCFSRYPDHMKRHDFFKSAMPEGYVQERTIYFKDDGTYKTRAEVKFEGDTLVNRIELKGIDFKEDGNILGHKLEYNFNSHKVYITADKQNNGIKANFTIRHNVEDGSVQLADHYQQNTPIGDGPVLLPGGRSREGRGSLLTCGDVEENPGPELMVSKGEEDNMAIIKEFMRFKVHMEGSVNGHEFEIEGEGEGRPYEGTQTAKLKVTKGGPLPFAWDILSPQFMYGSKAYVKHPADIPDYLKLSFPEGFKWERVMNFEDGGVVTVTQDSSLQDGEFIYKVKLRGTNFPSDGPVMQKKTMGWEASSERMYPEDGALKGEIKQRLKLKDGGHYDAEVKTTYKAKKPVQLPGAYNVNIKLDITSHNEDYTIVEQYERAEGRHSTGGMDELYK*

**Supplementary Figure S1.** Sequence of biosensor used in this study. Color of text is correspond to those from schematic presentation of primary structure shown in main figure.

**Supplementary Figure S2.** Detection of HCoV-OC43 infection by FlipGFP system. (A) Primary structure of 3CLpro-FlipGFP biosensor. ORF of GFP β10: G10 (pink), coiled-coil dimerization sequence E5 (black), GFP β11: G11 (light blue), coiled-coil dimerization sequence K5: K5 (black), T2A self-cleaving peptides (white), GFP 1-9: GFP β1-9 (light green), mCherry (orange), an internal control, are indicated. (B) GFP fluorescens in cells expressing 3CLpro-FlipGFP biosensor. 293T cells stably expressing 3CLpro-FlipGFP were transfected with Myc-tagged SARS-CoV-2 3CL protease or infected with HCoV-OC43 at MOI = 1.0, then cells were harvested at 48 h post-transfection or post-infection. Means fluorescence intensity (MFI) of GFP are measured by flow cytometer.

**
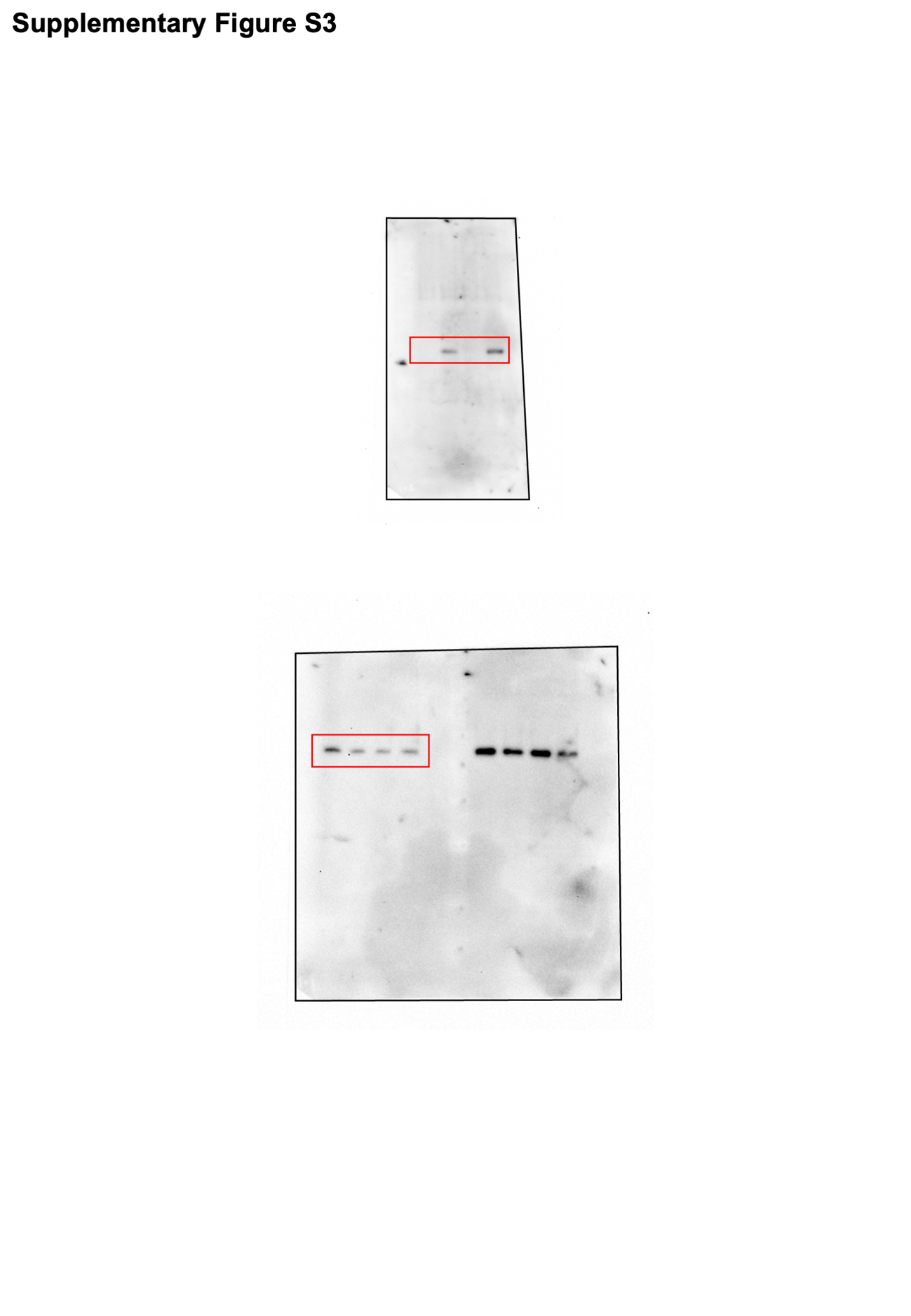
**

**Supplementary Figure S3.** Original westernblotting pictures for those presented in main text. Those for Figure 3B are shown. Upper panel is for anti-Myc antibody western blotting, lower panel is for anti-a-Tubulin antibody western blotting.

**
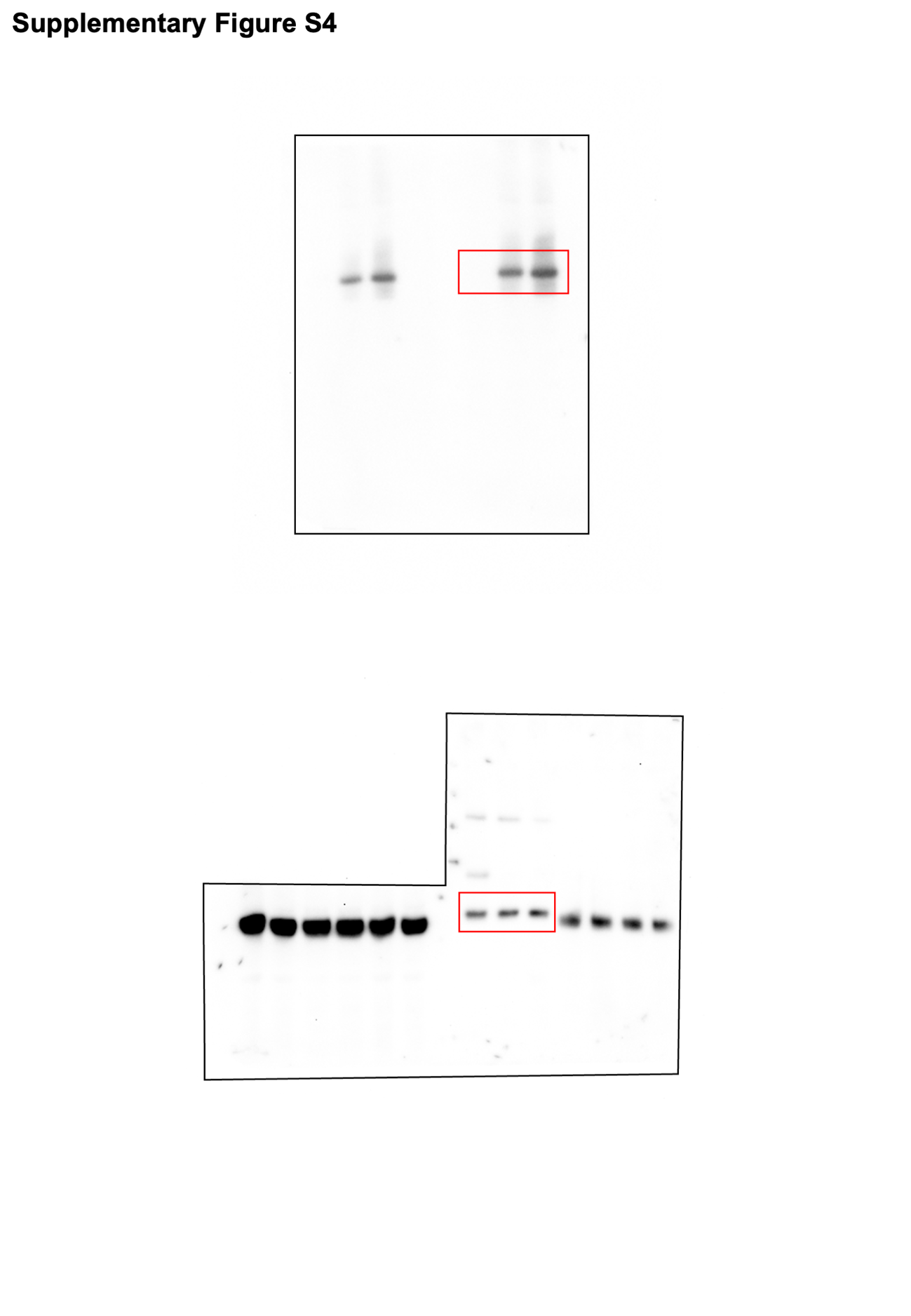
**

**Supplementary Figure S4.** Original western blotting pictures for those presented in main text. Those for Figure 4B are shown. Upper panel is for anti-Myc antibody western blotting, lower panel is for anti-a-Tubulin antibody western blotting.


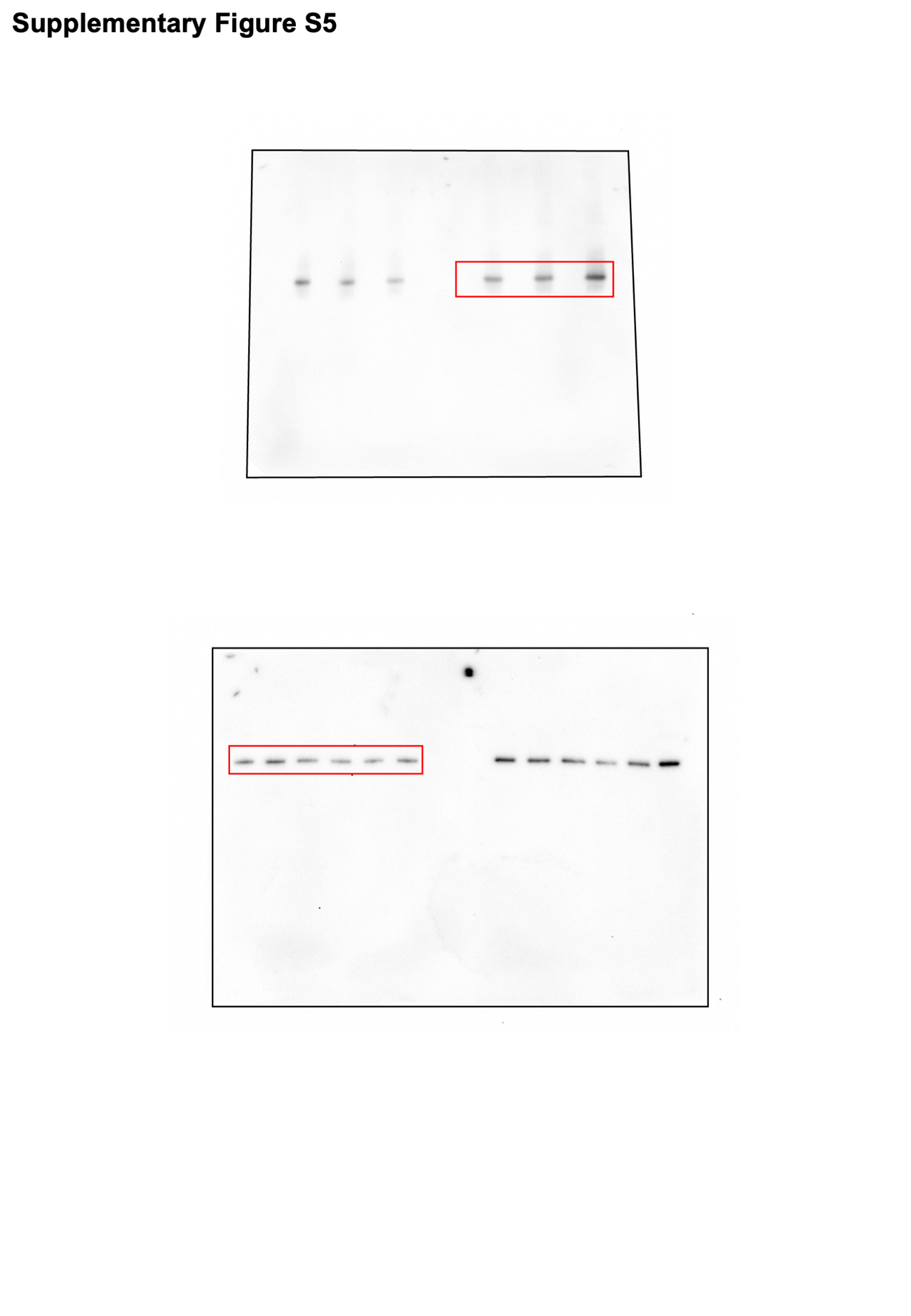


**Supplementary Figure S5.** Original westernblotting pictures for those presented in main text. Those for Figure 5C are shown. Upper panel is for anti-Myc antibody western blotting, lower panel is for anti-a-Tubulin antibody western blotting.
